# Effects of mindfulness‐based interventions on fatigue and psychological wellbeing in women with cancer: A systematic review and meta‐analysis of randomised control trials

**DOI:** 10.1002/pon.6046

**Published:** 2022-10-13

**Authors:** Kairen McCloy, Ciara Hughes, Lynn Dunwoody, Joanne Marley, Jackie Gracey

**Affiliations:** ^1^ Ulster University Newtownabbey UK

**Keywords:** cancer, meta‐analysis, mindfulness, mindfulness‐based cognitive therapy, mindfulness‐based stress reduction, oncology, psycho‐oncology, systematic review, women

## Abstract

**Background:**

Cancer diagnosis and treatment can cause fatigue, stress and anxiety which can have a detrimental effect on patients, families and the wider community. Mindfulness‐based interventions appear to have positive effects on managing these cancer‐related symptoms.

**Objective:**

To investigate the efficacy of mindfulness on cancer related fatigue (CRF) and psychological well‐being in female cancer patients.

**Methods:**

Five databases (CINHAL, Ovid Medline, Ovid Psych Info, Scopus, and Cochrane), and two trial registers (WHO and Clinicaltrials.gov) were searched for randomised control trials from inception to April 2021 and updated in August 2022. Meta‐analysis was performed using Review Manager 5.4. The standardised mean difference (SMD) and 95% confidence intervals (CI) were used to determine the intervention effect. Subgroup analysis was performed for adaptation to types of mindfulness, length of intervention and types of comparator used.

**Results:**

Twenty‐one studies with a total of 2326 participants were identified. Mindfulness significantly improved CRF (SMD −0.81, 95% CI −1.17 to −0.44), depression (SMD−0.74, 95% CI −1.08 to −0.39) and anxiety (SMD −0.92, 95% CI −1.50 to −0.33). No effect was observed for quality of life (SMD 0.32, 95% CI −0.13–0.87) and sleep (SMD −0.65, 95% CI −1.34–0.04). Subgroup analysis revealed that there was little difference in SMD for adapted type of mindfulness (*p* = 0.42), wait list control compared to active comparator (*p* = 0.05) or length of intervention (*p* = 0.29).

**Conclusion:**

Mindfulness appears to be effective in reducing CRF and other cancer related symptoms in women. Adaptations to mindfulness delivery did not have negative impact on results which may aid delivery in the clinical settings.

## 1 BACKGROUND

Worldwide there are an estimated 8.5 million women with cancer, while in the UK there are more than 182,000 new cases per year.^1^, ^2^ Symptoms associated with diagnosis and treatment of cancer include depression, anxiety, sleep deprivation and fatigue. Cancer‐related fatigue (CRF) prevalence is higher in females and it is rated as the fourth most common unmet need among those living with and beyond gynaecology cancer.^3^, ^4^, ^5^, ^6^

The definition of CRF provided by the National Comprehensive Cancer Network illustrates its pervasiveness, as it is deemed to be a persistent feeling of tiredness that is physical and cognitive, it is not related to activity level and is not relieved by sleep.^7^ CRF is multifaceted in nature and presentation, hence the interventions to manage this symptom need to be reflective of this. However, currently, the advice to manage CRF continues to be keeping active, eating a healthy diet and sleep hygiene.^8^ However, research suggests that interventions such as exercise have mixed results for managing CRF, with some studies showing little or no effect.^9^, ^10^, ^11^, ^12^, ^13^, ^14^ Furthermore, it seems that exercise alone may not successfully target all aspects of CRF, for example, emotional or cognitive fatigue, may actually hinder participants' engagement in interventions such as exercise.^15^, ^16^, ^17^ Diet and sleep hygiene although have evidence that may impact CRF, there remains a lack of studies that incorporate the role of diet in the direct management of CRF or sleep interventions that improve sleep substantially.^18^ This lack of improvement in sleep can directly or indirectly effect CRF, this is known as a phenomena called ‘cluster symptoms’ where more than one symptom cluster together and effect each other positively or negatively, for CRF the symptoms identified include sleep, anxiety and depression.^19^ The presence of these symptoms can lead to stress which may also lead to a further increase in the cluster symptoms.^20^ The reduction of stress for cancer patients may be a factor in enhancing the management of CRF and interventions that can have the mind‐body impact that may result in improvements in lifestyle include interventions like mindfulness.

Mindfulness has been described as the intention of being aware of the present moment without judgement.^21^ And the research for cancer in this area has seen exponential growth over the last 10 years. However, the majority of this research has focused on reduction of depression, anxiety and stress symptoms associated with CRF.^22^, ^23^, ^24^, ^25^ Practising mindfulness has been shown to help emotional self‐regulation, the development of positive coping mechanisms, and stress reduction, leading to improved quality of life (QoL) in women with breast cancer.^26^, ^27^ Mindfulness alone may be helpful in managing CRF or it can be the starting point that will allow individuals to access interventions that may further enhance the management of CRF.

Previous systematic reviews have evaluated the impact of mindfulness on psychological wellbeing,^28^, ^29^, ^30^ showing a moderate effect on reducing anxiety and depression. However, some of these reviews were restricted in terms of the types of mindfulness for example, mindfulness‐based stress reduction (MBSR) or mindfulness‐based cognitive therapy (MBCT), and others only included certain cancer population, for example, breast cancer.^28^, ^29^, ^31^, ^32^ The reviews that evaluated mindfulness in relation to CRF have also focused on specific types of mindfulness or have only included certain populations or types of cancer.^33^, ^34^ However, a recent review^34^ reported the positive effects that mindfulness had on both CRF and vitality, the authors of this review assessed CRF as tiredness and exhaustion and vitality as energy and levels of function, each was evaluated separately. This review included any type of cancer, males and females, at any stage of cancer and any type of mindfulness. To date, no systematic review has evaluated research studies that included women with cancer, any type of mindfulness and its impact on CRF. Hence, the aim of this review was to assess evidence for the impact of any type of mindfulness on CRF in women with cancer. The secondary aim was to consider the impact of mindfulness on psychological wellbeing, which was defined in the current review as depression, anxiety, and sleep, all of which are described as part of the symptom cluster of CRF.

## 2 METHOD

### 2.1 Search strategy

This review followed the preferred reporting of items of systematic reviews and meta‐analysis (PRISMA) statement.^35^ The protocol was registered in PROSPERO CRD42021240439.^36^ A systematic search was conducted using the Cochrane Central Register of Controlled Trials (CENTRAL), the Cochrane Database of Systematic Reviews, Ovid MEDLINE(R), EBSCO Cumulative Index to Nursing and Allied Health Literature (CINAHL), Ovid PsycINFO and, Scopus from inception until April 2021 and updated in August 2022. Trials registries were also searched, clinicaltrials. gov and WHO ICRTP along with Open Grey for grey literature. Hand searching and citation searching were also performed of eligible studies to identify any missing studies that databases may have missed. Each database was searched with a combination of MeSH and Keywords (1) *cancer* or *neoplasm,* (2) *fatigue* or *tiredness* or *lethargy, and* (3) mindfulness or *meditation.* (Additional search strategy provided in supporting material).

### 2.2 Inclusion and exclusion criteria

Studies were eligible for inclusion if they were randomised control trials (RCT) testing a mindfulness based intervention (e.g., MBSR, mindfulness‐based cognitive therapy (MBCT), mindfulness‐based art therapy (MBAT)), participants were female, over 18 with a diagnosis of cancer, fatigue was measured at baseline and one other timepoint post intervention, and studies were published in English. Comparison groups included treatment as usual/waitlist control or active treatments such as supportive care or education. Studies were excluded if mindfulness was not the main component (e.g., Acceptance and Commitment Therapy) and if reported as poster and or conference reports or abstracts.

### 2.3 Study selection

Studies were initially screen by titles and abstracts and excluded if they did not focus on fatigue and women with cancer. Full text articles were retrieved and checked for eligibility, by a team of reviewers and any disagreements resolved by consensus. RefWorks bibliography software was used to export, manage and deduplicate search results, with additional hand removal of duplicates.

### 2.4 Data extraction

Data were individually extracted (KMCC) and checked, by a team of reviewers, and any discrepancies were resolved through discussion. Data extracted included: author, year of publication, country of origin, age, stage of cancer, type of cancer, treatment status, intervention arms, outcomes, measures, eligibility criteria, assessment timepoints, the results such as the mean and standard deviations (effect size) for CRF, anxiety, depression, sleep and QoL and adverse events. An Excel spreadsheet was designed to capture these data.

### 2.5 Risk of bias assessment

The risk of bias was assessed for all 23 using the Cochrane risk‐of‐bias tool for randomised trials (RoB 2)^37^ which includes 5 domains: randomisation, deviations from interventions, outcome measures, missing outcome data, reporting of results all of which are judged as low, some concerns and high with a summarised overall risk. This was completed by (KMCC) and 21% independently checked by other reviewers any discrepancies were discussed and consensus reached within the team.

### 2.6 Quality of assessment

The Grading of Recommendations, Assessment, Development and Evaluation (GRADE) was performed to assess the quality of evidence of the included studies. This assessment goes beyond the risk of bias and includes 5 domains for assessment: risk of bias, inconsistency of the results, indirectness, imprecision, and publication bias possible ratings include high, moderate, low and very low.^38^ GRADEpro was used to perform this assessment, and data for each outcome generated a ‘Summary of Findings Table’ with associated footnotes that explained any decisions on downgrading of the quality of evidence.

### 2.7 Data analysis

Review Manager software (RevMan version 5.4) was used to conduct the meta‐analysis, using a random‐effects (inverse variance method) as heterogeneity in treatment effects was anticipated due to between‐study variations in clinical factors (e.g., content of intervention).^39^ Effects sizes were calculated as standardised mean differences (SMDs) with 95% confidence intervals (CIs) indicating the difference in means between groups divided by the pooled standard deviation (SD). Effect sizes were categorised by Cohen's classifications: SMD 0.2–0.5 small effect, SMD 0.5–0.8 medium effect, and SMD >0.8 large effect.^40^ A negative SMD for CRF, depression, anxiety and sleep indicated a larger improvement in these outcomes due to the mindfulness intervention. For QoL a positive SMD indicated a larger reduction. Heterogeneity was evaluated using the *I* ^2^ statistics with values of 0%–40% representing might not be important, 30%–60% representing moderate, 50%–90% representing substantial and 75%–100% considerable heterogeneity.^41^ Data was extracted for pre, post and first follow up for both intervention and control groups. Effect sizes were calculated for pre to post‐treatment and for pre‐treatment to last follow up.

### 2.8 Subgroup analysis

Subgroup analysis was intended for types of intervention (MBSR *v* non‐MBSR), length of intervention (8 weeks *v* > than 8 weeks) and active comparator (AC) versus wait‐list control (WLC) and usual care (UC).

### 2.9 Addressing missing data

In those studies that did not provide all the required data, authors were contacted via email to request SDs and means. If it was possible to calculate this from other data provided such as standard error (SE), CIs, or *t*‐values, then this was performed. Where it was not possible to access SDs and mean, these were excluded from the meta‐analysis.

### 2.10 Sensitivity analysis

Sensitivity analysis was carried out to ensure the robustness of the effect. For studies with a high risk of bias, all removal of these studies was performed to determine sensitivity to the results. If substantial or considerable heterogeneity was evident while performing meta‐analysis, then sensitivity analysis was also performed to explore reasoning. This may be performed by removing outliers with effect sizes that are two standard deviations from the pooled effect size.^42^

## 3 RESULTS

### 3.1 Studies included

**FIGURE 1:**
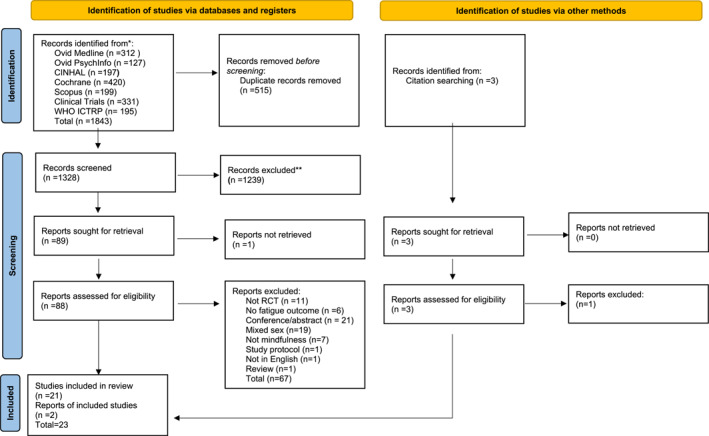
PRISMA flow diagram

Electronic databases searching identified 1843 records. After deduplication, 1328 records were screened using the title, and the abstracts of these 1239 were excluded. This left 89 records, of which one report was unable to be retrieved. Following screening of the full text of 88 records 67 were excluded leaving 21 publications searching of reference lists and forward citations found, 2 additional eligible papers, resulting in a total of 23 eligible papers. Of the 23 papers, 20 were original RCT studies, and 3 were secondary publications of studies already included. Of the 23 records examined, 20 included sufficient evidence for analysis (Figure 1 PRISMA flow diagram).

### 3.2 Study characteristics

A total of 2326 participants were identified with an average of 116 per study (range 24–322). The mean age of the sample was 51 (range 41–64). The percentage of participants with breast cancer was 97%, and the remaining were gynaecology cancer (0.95%), Heamatology malignancy (0.65%), neuro‐oncology (0.25%), rectal carcinoma (0.30%) and other (0.85%). Most studies (*n* = 8, 42%)^43^, ^44^, ^45^, ^46^, ^47^, ^48^, ^49^, ^50^, ^51^, ^52^ adopted MBSR as the type of mindfulness programme. For many of the studies the intervention was greater than 8 weeks (*n* = 15, 79%)^43^, ^47^, ^48^, ^49^, ^50^, ^51^, ^53^, ^54^, ^55^, ^56^, ^57^, ^58^, ^59^, ^60^, ^61^, ^62^ duration. The comparator used was mainly (*n* = 11, 52%)^43^, ^44^, ^45^, ^46^, ^49^, ^52^, ^55^, ^56^, ^58^, ^60^, ^61^, ^62^, ^63^ waitlist control or UC with only (*n* = 8, 38%)^47^, ^48^, ^50^, ^51^, ^53^, ^54^, ^57^, ^59^, ^64^ using an AC. Only one study^49^ assessed fatigue at baseline and stated fatigue severity score as an inclusion criteria. Very few (*n* = 5, 25%)^43^, ^51^, ^57^, ^60^ reported adverse events, and those studies that did report stated that there were no adverse events (Table 1).

**TABLE 1:** Table of characteristics

		Sample size		Mean age		Race & ethnicity			Stage of cancer			Intervention features	
Author year	Fatigue eligibility criteria	MBI	Control	MBI	Control	MBI	Control	Type of cancer	MBI	Control	Origin	MBI (type)	Control
Bower 2015	N/A	39	32	46.1 (28.4–60)	47.7 (31.1–59.6)	White 29, African American1, Asian 5, other 1	White 25, African American 1, Asian 5, other 1	Breast	NR	NR	USA	(MAP) 6 weeks, 2 h per week	Wait list control.
Bower 2021	N/A	85	81	44.5 (7.7)	45.8 (5.6)	White 75, black 3, Asian 5, other 2, hispanic 10	White 126, black 16, Asian 15, other 3, hispanic 14	Breast	NR	NR	USA	(MAP) 6 weeks, 2 h per week	SE 6 weeks
Carlson 2013 & 2006	N/A	134	118	55.12 (9.84)	54.14 (10.23)	NR	NR	Breast	0–5 (3.7), I‐56 (41.8), II‐51 (38.1), III‐17 (12.7), IV‐1 (0.7), Unkown‐4 (3.0)	(N9%) 0–2 (1.7), I‐49 (41.5), II‐42 (35.6), III‐16 (13.6), IV‐2 (1.7), Unknown‐7 (5.9)	Canada	MBSR 8 weeks group sessions, 90 min each	SET 12 weeks 1.5 h each. SMS 1 day
Chu 2020	N/A	42	42	54.6 (5.7)	54.9 (6.3)	NR	NR	Breast	NR	NR	China	MBCT 8 weeks, 2 h per week	Usual care
Dodds 2015	N/A	16	17	54.7 (12.1)	55.8 (9.7)	White 11 (92), non‐white 1 (8)	White 12 (75), non‐white 4 (25)	Breast	I‐3 (25), II‐5 (42), III‐4 (33), IV‐0 (0)	I‐1 (6), II‐9 (56), III, 4 (25), IV‐2 (13)	USA	CBCT 8 weeks, 2 h classes	Wait list control
Franco 2019	N/A	19	17	41.27 (9.76)	Not reported	NR	NR	Breast	NR	NR	Spain	Flow meditation 7 weeks, 2 h per week	Not stated
Gok Metin 2019	N/A	32	31 (PMR) 29 (CG)	48.21 (10.23)	PMR 46.67 (10.06) CG 52.86 (11.70)	NR	NR	Breast	I‐4 (12.5), II‐16 (50), III‐12 (37.5)	PMR‐I‐4 (12.9), II‐19 (61.3), III‐8 (25.8) CG I‐1 (4), II‐16 (55.2), III‐12 (41.4)	Turkey	Mindfulness meditation daily once a day for 12 weeks	PMR (progressive muscle relaxation) daily once a day for 12 weeks
Hoffman 2012	N/A	12	12	49 (9.26)	50.1 (9.14)	NR	NR	Breast	0–11 (10), 1–34 (30), II‐47 (41), III‐22 (20)	0–6 (5), I‐45 (39), II‐47 (41), III‐17 (15)	UK	MBSR 8 weeks 2 h per week, first session 2.25 h	Wait list control
Jang 2016	N/A	12	12	51.75 (5.32)	51.42 (6.33)	NR	NR	Breast	NR	NR	Korea	MBAT 12 weeks 45 min each	Wait list control
Kenne 2017	N/A	62	52 (active controls) 52 (non‐MBSR)	57.2 (10.2)	57.2 (10.2)	NR	NR	Breast	NR	NR	Sweden	MBSR 8 weeks 2 h per week	Active control‐8 weeks self‐instruct MBSR, CG
Lengacher 2009 & 2012	NA	41	43	57.1	58.0	White 36 (87.8), hispanic 6 (14.6), black‐ 2 (4.9) other 3 (7.3)	White 34 (79.1), hispanic 3 (7), black 8 (18.6), other 1 (2.3)	Breast	0–5 (12.2), I‐26 (63.4), II‐7 (17.1), III‐3 (7.3)	0–9 (20.9), I‐19 (44.2), II‐12 (27.9), III‐3 (7)	USA	MBSR 6 weeks, 2 h per week.	Wait list control
Mattes 2019	N/A	10	15	64.2 (9.1)	62.9 (9.1)	NR	NR	Breast	Stage I‐III no breakdown	Stage I‐III no breakdown	Germany	MBSR 8 weeks	Nordic walking 8 weeks
Monti 2006	N/A	56	55	53.1 (12.4)	54.1 (10.7)	Caucasian 45 (80), African American 10 (18), Asian 1 (2), hispanic 0,	Caucasian 38 (69), African American 13 (24), Asian 1 (2), hispanic 2 (4), other 1 (2)	Breast 51 (46), gynaecology 19 (17), haematology 13 (12), neuro 5 (5), rectal 6 (5), other 17 (15)	29−0,I, II, 27‐III, IV	28−0, I, II, 27‐III, IV	USA	MBAT 8 weeks, 2.5 h	Wait list control
Monti 2013	N/A	98	93	56.9 (12.4)	56.4 (10.7)	Caucasian 59 (60), African American 35 (36), Asian/Other 0	Caucasian 54 (58), African American 37 (40), Asian/Other 2 (2)	Breast	0‐I (1), I‐36 (37), II‐25 (26), III‐11 (11), IV‐3 (3), unkown‐22 (22)	0–1 (1), I‐39 (42), II‐23 (25), III‐5 (5), IV‐2 (2), unkown‐23 (2)	USA	MBAT 8 weeks	8 weeks meeting weekly.
Park 2020	N/A	38	36	53.21 (8.4)	54.19 (9.27)	N/A	N/A	Breast	0–9 (23.7), I‐13 (34.2), II‐14 (36.8), III‐2 (5.3)	0–6 (16.7), I‐14 (38.9), II‐15 (41.7), III‐1 (2.7)	Japan	MBAT 8 week, 2 h per week	Wait list control
Rahmani 2014	NR	12	12 (MCT) 12 (UC)	43.25 (3.08)	MCT 44.92 (1.83) UC 44.92 (1.8)	NR	NR	Breast	NR	NR	Iran	MBSR 8 weeks, 2 h sessions per week	Metacognition (MCT) 8 sessions.
Rahmani 2015	Fatigue severity at baseline	12	12	43.25 (3.07)	44.8 (28)	NR	NR	Breast	NR	NR	Iran	MBSR 8 weeks, 2 h sessions	None
Reich 2017 & Lengacher 2016	N/A	167	155	56.5 (10.2)	57.6 (9.2)	Caucasian 112 (67.1), black 21 (12.6), hispanic 19 (11.4), other 15 (9)	Caucasian 110 (71.0), black 16 (10.5), hispanic 14 (9.1), other	Breast	0–21 (12.6), I‐53 (31.7), II‐61 (36.5), III‐32 (19.2)	0–19 (12.3), I‐56 (36.1), II‐54 (34.8), III‐26 (16.8)	USA	MBSR (BC) 6 weeks 2 h per week	Usual care
Van der gucht 2020	N/A	18	15	43.89 (6.03)	47.4 (5.45)	NR	NR	Breast	NR	NR	Belgium	MBSR/MBCT 8 weeks × 4 3 h sessions	Wait list control
Witek Janusek 2019	N/A	96	96	55 (10.1)	55.2 (10.1)	Caucasian 80.8, African American 11.5, hispanic 2.6, PI/Asian 1.3, other 1.3	Caucasian 72.5, African American 15.8, hispanic 6.6, PI/Asian 1.3, other 2.6	Breast	0–22.9, I‐51.8, II‐16.9, III‐8.4,	0–24.4, I‐62.2, II‐11.0, III‐2.4	USA	MBSR 8 weeks 2.5 h per week	Active control condition (ACC) 8 week

Abbreviations: CBCT, Compassion based cognitive therapy; MAPS, mindfulness awareness practice; MBAT, mindfulness‐based art therapy; MBCT, mindfulness‐based cognitive therapy; MBSR, mindfulness‐based stress reduction; MBSR (BC), mindfulness‐based stress reduction breast cancer; MCT, metacognition therapy; PMR, Progressive muscle relaxation; SET, Supportive Expressive therapy; SE, Survivorship Education; SMS, Stress management seminar.

### 3.3 Pooled effects at post‐intervention

**FIGURE 2:**
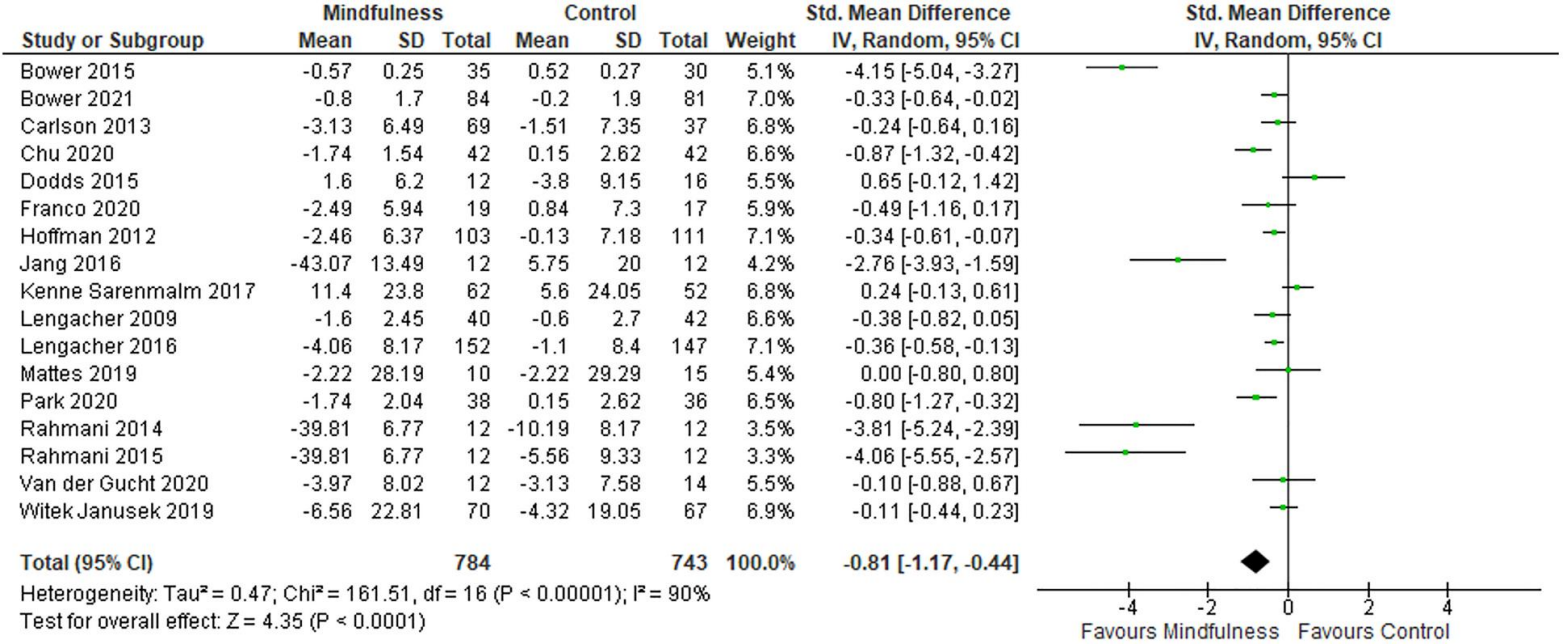
Forest Plot for fatigue‐post intervention effect size

A forest plot for effect size of CRF is shown in Figure 2 17 studies were included in this analysis. As shown in Table 2 mindfulness significantly improved CRF post‐intervention with the effect size being large (SMD −0.81). In addition, mindfulness had a significant effect on the secondary outcomes of depression and, anxiety with effect sizes being large (depression; SMD −0.74; anxiety; SMD −0.92). However, mindfulness did not have a significant effect on both QOL and sleep.

**TABLE 2:** Outcomes measures pooled effects for post intervention and follow up

Outcome	Sample size		Effect size^a^			Heterogeneity^b^	
Post‐intervention:	K	N	SMD	95% CI	*p*	I^2^	*p*
Fatigue	17	1527	−0.81	−1.17 to −0.44	**<0.0001**	90%	<0.00001
Anxiety	7	1017	−0.92	−1.50 to −0.33	**0.002**	95%	<0.00001
Depression	12	1347	−0.74	−1.08 to −0.39	**<0.0001**	88%	<0.00001
QOL	10	925	0.37	−0.13 to –0.87	0.15	91%	<0.00001
Sleep	5	597	−0.65	−1.34 to 0.04	0.06	92%	<0.0001
Follow‐up							
Fatigue	12	1141	−0.55	−0.86 to −0.25	**0.0003**	80%	<0.00001
Anxiety	4	675	−0.59	−0.93 to −0.25	**0.007**	76%	0.006
Depression	8	1052	−0.28	−0.58 to 0.01	0.06	80%	<0.00001
QOL	8	791	−0.06	−0.65 to 0.53	0.85	92%	<0.00001
Sleep	4	504	0.04	−0.47 to 0.55	0.89	83%	0.0006

*Note*: Bold values indicate statistical significance.

Abbreviation: QOL, Quality of life.

^a^ ES = SMD, fatigue, anxiety, depression and sleep negative values indicates a favourable response. For QOL a positive value indicates a positive result. Values: small (0.2–0.5), moderate (0.5–0.8), large (>0.8).

^b^ *p* values <0.1 taken to suggest heterogeneity. *I* ^2^ statistics: 0% (no heterogeneity), 25% (low heterogeneity), 50% (moderate heterogeneity), and 75% (high heterogeneity).

There was evidence of considerable heterogeneity between studies for change in CRF (*p* = <0.00001, *I* ^2^ = 90%) depression (*p* = <0.00001, *I* ^2^ = 88%), anxiety (*p* = <0.00001, *I* ^2^ = 95%) QoL (*p* = <0.00001, *I* ^2^ = 91%) and sleep (*p* = <0.00001, *I* ^2^ = 92%). Sensitivity analysis was performed by removing outliers, which reduced the heterogeneity for CRF, anxiety and sleep from considerate to moderate or not being present, but did not appear to change the levels of heterogeneity for depression or QoL (See Table 2). The removal of outliers also reduced the pooled effect size from large to between moderate or small for all outcomes. Further sensitivity analysis was performed by removing studies with poor study quality which resulted in a reduction in heterogeneity from considerate to where it may not be considered important for CRF, anxiety and sleep but resulted in little change in heterogeneity for depression. Although the pooled effect size was also reduced from large to moderate or small for all outcomes (Table 2).

### 3.4 Sub‐group analysis

Sub group analysis was performed for type the of mindfulness, length of intervention and comparator used.

The effects of the mindfulness intervention were analysed as two groups, those studies that used MBSR as the mindfulness intervention compared with studies that used other types of mindfulness that were non‐MBSR (MBCT, MBAT, MBCR, Flow and CBCT). Both groups showed positive effect of mindfulness on CRF (non‐MBSR; SMD −0.93; MBSR; SMD −0.62) but there was no significant differences between groups (*p* = 0.42). There were significant differences for depression and anxiety, with non‐MBSR showing a greater pooled effect than MBSR (Table S1 in supporting materials for further information). There were no significant sub‐group differences for QoL or sleep, however given that there was only one study in the non‐MBSR sleep group, this sub‐group analysis result should be interpreted with caution.

Heterogeneity remained considerable for sub‐groups in the outcome of fatigue, depression, anxiety and QOL. For depression and anxiety the level of heterogeneity was reduced to low and very low in the MBSR group within the sub‐group analysis (Supporting materials Table S1).

In terms of length of intervention, studies of 8 weeks duration in length were compared to those that were less than 8 weeks. One study was of 12 weeks duration as this was considered an outlier, it was omitted from the analysis. For CRF, anxiety, and depression, there appeared to be no significant difference between sub‐groups with both showing a favourable response to mindfulness (SMD range −0.56 to −1.03). Mindfulness did not have a significant effect on QOL and sleep (Table S1 in supporting materials).

In the comparator analysis studies that used a WLC or UC were grouped together, and studies that used what was defined as AC were grouped together. For CRF, and anxiety this analysis indicated no significant difference between groups however, the pooled effect and overall effect showed a favourable response to the WLC group (CRF WLC; *p* = <0.0001 anxiety WLC; *p* = 0.03) with little or no effect for AC groups (CRF AC *p* = 0.13; anxiety AC *p* = 0.08) heterogeneity remained considerable for both these outcomes. Although there was a statistical significant between groups for depression the favourable response like the other outcomes, of CRF and anxiety, remained large for WLC (SMD −1.14) whereas AC showed little or no effect and demonstrated no heterogeneity within in this analysis (Table S1 in supporting materials). The subgroups for depression differed in the number of studies and number of participants suggesting that subgroup analysis may not be able to detect differences. For QOL and sleep both groups did not show a favourable response to mindfulness.

### 3.5 Pooled effects at follow up

Only (*n* = 12) studies provided data that could be included in an analysis Table 2 shows mindfulness^43^, ^45^, ^47^, ^49^, ^50^, ^51^, ^52^, ^55^, ^56^, ^60^, ^61^, ^63^ continued to show a significant pooled effect for CRF at follow up (SMD −0.55) and for the secondary outcomes of depression and anxiety (depression; SMD −0.28; anxiety; SMD −0.59). For QoL, and sleep the follow up remained non‐significant and continued to show little or no effect. The follow up periods for the studies included in this analysis were between 4 weeks and 6 months this variation along with the differing characteristics of the included types and durations of mindfulness may have contributed to the considerable heterogeneity that was shown in the analysis (Table 2).

### 3.6 Risk of bias

**FIGURE 3:**
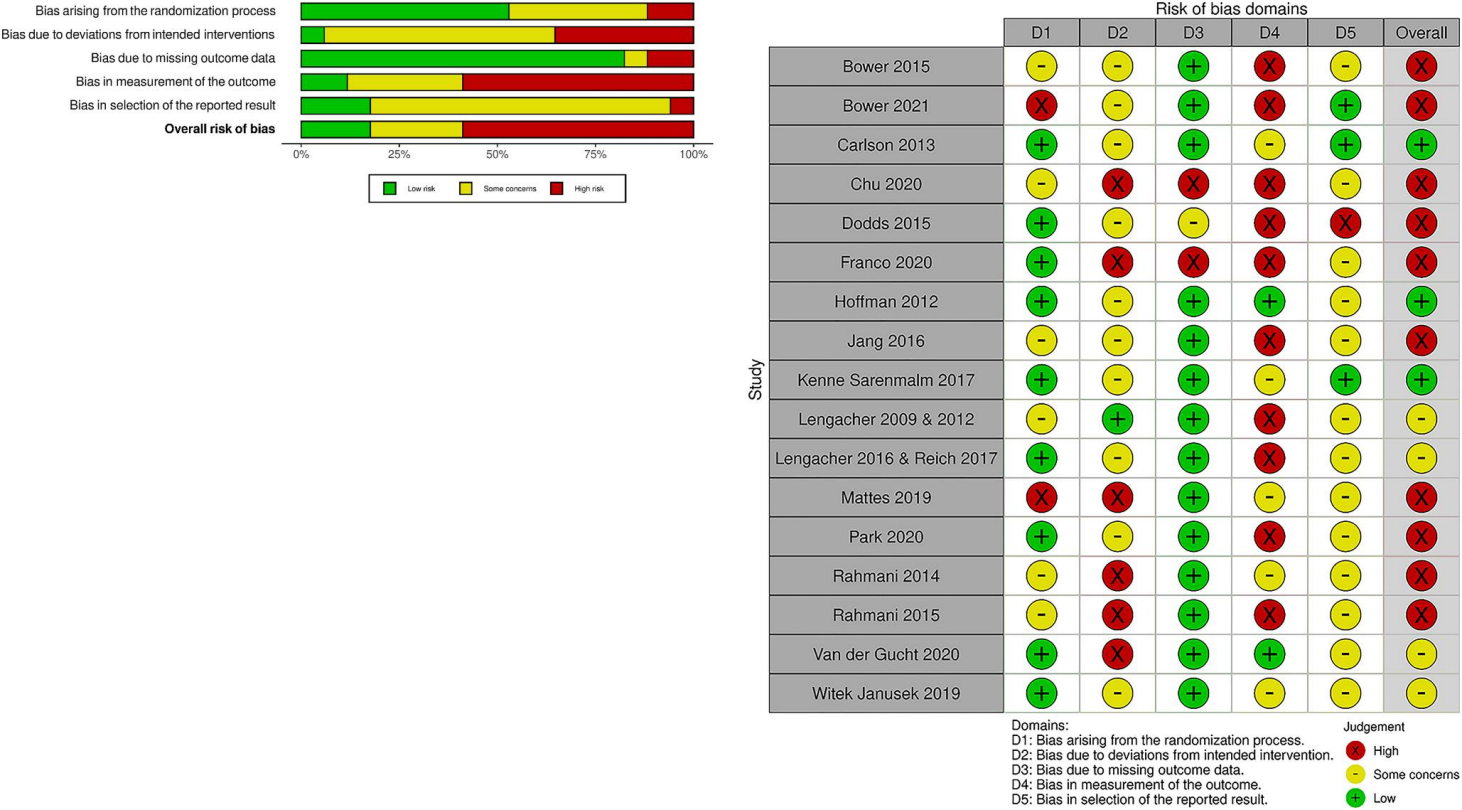
Risk of bias for fatigue outcome

Results indicated that over 50% of studies were, assessed as having high overall risk of bias, mainly due to lack of blinding associated with outcome measures (domains 4) and deviations from intended interventions (domain 2). However, it must be noted due to the nature of these intervention studies, blinding is a common issue as it is difficult to blind participants and outcome assessors to interventions as quite often the outcomes are patient reported. The domain with the lowest risk of bias assessed missing outcome data (domain 3), with 14 out of the 20 studies assessed as low. This was expected, as most of the studies accounted for missing data through intention‐to‐treat analysis. Risk of bias from the randomisation process was also low (domain 1), as most studies provided adequate descriptions, however, many of the studies failed to adequately describe the allocation. The level of some concern was also high for reporting of studies, as very few trials were either pre‐registered or had a pre‐defined analysis plan (See Figure 3).

### 3.7 Grading of recommendations, assessment, development and evaluation

Using GRADE the overall quality of evidence was rated as low or very low suggesting a low level of confidence in the effect estimate. The level of evidence for RCT was downgraded from high to low for sleep and anxiety and very low for fatigue, depression and QoL. This reduction from high to low or very low was due to serious concerns regarding levels of heterogeneity, risk of bias and publication bias. Overall, no serious concern were found for inconsistency or indirectness (See Table S2 Summary of findings table in supporting materials).

### 3.8 Publication bias

Publication bias was assessed using funnel plots for fatigue and, QoL, outcome measures that have fewer than 10 studies were not assessed as a minimum of 10 studies is required for a funnel plot to detect bias.^65^ The funnel plots demonstrated asymmetry which would be suggestive of publication bias (Figure S1 in supporting material).

## 4 DISCUSSION

To our knowledge this is the first study to conduct a meta‐analysis on the impact of mindfulness on fatigue for women with cancer. The results of this meta‐analysis suggest that mindfulness led to a reduction in CRF post intervention and at follow up. For the secondary aim, mindfulness reduced anxiety and depression post intervention, with slight reductions at follow up, however no significant improvements were noted in sleep or QoL. The analysis showed that there was considerable heterogeneity between studies for all outcomes an attempt to account for this was performed through sensitivity analysis and removal of outliers although there was some reduction it continued to be considerable (Table S1 in supporting material).

This large effect of mindfulness on CRF (SMD −0.81) is in keeping with other reviews that also demonstrated similar pooled effects for CRF (Xie 2020)^33^ (SMD −0.89), Zhang 2016^66^ (SMD −0.88). However, in contrast, some reviews have demonstrated a smaller or even a larger pooled effect size than the current review demonstrated, regardless, these reviews have all shown a favorable response for mindfulness in improving CRF (Lin et al. 2022),^67^ (SMD −0.56), Zhang et al. 2019^29^ (SMD −0.66), Haller et al. 2017^31^ (SMD −0.28), Xunluin et al. 2020.^30^ (SMD 0.48) Schell et al. 2019^32^ (SMD −0.50). Indeed in a recent meta‐analysis,^68^ that examined the effect of psychosocial interventions on CRF demonstrated that MBSR had the largest impact when compared to other types of interventions. These conflicting results may have been due to differences in the types of mindfulness, types of cancer, outcomes and aims within these reviews. Reviews on mindfulness and CRF have to date focused on either a specific type of mindfulness (MBSR) or specific cancers, such as breast, additionally reviews that have included many types of mindfulness have included various types and stages of cancer.^32^, ^33^, ^34^ To date no review has assessed whether any type of mindfulness has an effect on CRF for women with cancer, therefore, this review has contributed new knowledge in this area.

Psychological well‐being, which included depression and anxiety, showed that mindfulness improved this symptom in this review. Many of the included studies had the primary aim of psychological well‐being.^43^, ^44^, ^45^, ^46^, ^48^, ^52^, ^53^, ^54^, ^55^, ^58^, ^59^, ^63^, ^69^ Improvement in the symptoms of anxiety and depression may directly or indirectly effect other symptoms, such as CRF or sleep. Indeed, Reich et al., (2017)^52^ examined the symptom cluster and identified that fatigue, sleep, and psychological well‐being were part of the cluster, suggesting they are interrelated and that they may impact each other. This interrelation of symptoms on CRF was also demonstrated in a mindfulness intervention of 249 women with breast cancer.^70^ Within this study, mindfulness had the biggest effect on CRF directly but also indirectly by relieving or preventing anxiety, depression and sleep disturbance. However, these relationships between CRF and sleep was not demonstrated in other research, Carlson and Garland, 2005,^71^ found a significant relationship between stress and fatigue which suggested that a reduction in stress affected fatigue. MBSR has been well documented for reducing and helping in the management of stress which may lead to an effect on fatigue, many of the studies in this review assessed this outcome as part of psychological well‐being.^44^, ^45^, ^46^, ^51^, ^52^, ^59^, ^63^ Other reviews also demonstrated an effect on depression and anxiety although the magnitude of the effect differed from large to small.^29^, ^30^, ^31^, ^66^, ^72^

Neither sleep nor QoL showed a significant effect when data were pooled for this review. This was also reflected in previous reviews, Zhang et al., 2016^29^ found women with breast cancer reported some improvement in sleep after a mindfulness intervention, but this was not statistically, which is in contrast to the findings of Cillessen et al., 2019.^72^ Few previous systematic reviews have assessed sleep as part of the review aims, indeed, within this review, only 5 studies post intervention and 4 at follow up assessed this as outcome, therefore the impact of mindfulness on sleep is difficult to interpret. Regarding QoL, previous systematic reviews have differed in the impact of mindfulness on this variable, with Cillessen et al. 2019,^72^ finding no significant impact, and Haller et al., 2017,^31^ reporting a small but significant effect. Within the current review the results there were no significant effects of mindfulness on QoL, however, the CI (SMD 0.37, 95% CI −0.13–0.87) was compatible with a slight improvement and little or no effect suggesting some improvement even though it was not statistically significant. Whether this was due to the variability of the outcome measures used in the included studies or other variables, it is difficult to ascertain, and further research for this symptom maybe warranted.

Few of the studies in this review assessed fatigue as a primary aim. Whilst Gok Metin et al., 2019,^57^ stated that fatigue was an outcome of primary interest, this study could not be included in the meta‐analysis, as the data presented within the paper did not allow for this, and the author was contacted but did not respond. This study did demonstrate a significant result for the reduction in fatigue following the interventions of progressive muscle relaxation or mindfulness meditation. It also suggested that the positive emotional effect of mindfulness may also have an effect on energy levels. Reviews that have attempted to examine the relationship of the effect of mindfulness with participants who have fatigue at base line have suggested a larger effect for participants that enter studies with fatigue than those studies that do not assess participant levels at entry.^73^, ^74^ Whether this is seen as an inevitable result as there may be more scope for improvement if participants pre scores are large for a symptom, or if mindfulness may truly be able to treat symptoms such as fatigue remains to be further investigated.

Studies that reported follow up at least at one time point after post‐intervention were analysed and showed that there was a continued favorable response for improvement for fatigue, depression and anxiety after a mindfulness intervention. However, the pooled effect was smaller at follow up than post intervention for all outcomes. Few reviews have examined the effects at follow up and those that did show the effect of mindfulness at follow up varied. For example, Cillessen et al., 2019,^72^ and Haller et al., 2017^31^ found that anxiety and depression continued to show improvement but CRF was not statistically significant. Johns et al., 2021,^34^ findings for CRF were similar to this review and continued to show effect for CRF but the effect size was smaller than at post intervention. These variations and some reduction in pooled effect sizes for outcome measures may be because some studies failed to report these data, but may also be a result of differences in the length of follow‐ups being, weeks or several months after the intervention.

Five studies made reference to their being no adverse events associated with their interventions, the remaining 75% of studies ignored this as part of their reporting. Even though the likelihood of adverse events for mindfulness is viewed as being low, this should be reported in all studies, as mindfulness can have a negative impact on people with anxiety disorders.^75^ Most of the included studies in this review had exclusion criteria associated with mental health contraindications for participating in mindfulness.^43^, ^44^, ^45^, ^46^, ^48^, ^49^, ^50^, ^51^, ^52^, ^53^, ^54^, ^55^, ^56^, ^57^, ^58^, ^59^, ^60^, ^61^ However, this may suggest that recruitment into mindfulness studies to ameliorate CRF may not be representative of the general cancer population and this has implications for clinical practice as discussed below.

### 4.1 Limitations

Among the strengths of this current review are its emphases on CRF and the inclusion of all types of mindfulness. This allowed for the inclusion of a wide variety of studies, and the focus on women with cancer alone also further gave rise to establishing knowledge in this area. However, the wide variety of studies may have resulted in considerable heterogeneity as the study intervention characteristics were many and varied. Attempts were made to explain this heterogeneity through sensitivity analysis and sub group analysis however, not all could be explained and may be a result of other variables that could not be accounted for. Also the review was limited to English which has excluded those studies from another language that may have been included.

### 4.2 Clinical implications

Future implications would include a more diverse population in terms of types and stages of cancer as well as ethnic diversity. Unfortunately, studies of this type tend to attract a particular participant profile that tends to be highly motivated. Within this review, the majority of participants were Caucasian (72.42%), had breast cancer (97.42%) and the level of education or socioeconomic background was difficult to establish, as few studies report these details. As this review was gender specific it was able to demonstrate the positive effects of mindfulness on outcomes for women but also highlighted that other types of female cancer such as gynaecology cancer (0.95%) are underrepresented within the literature. Female cancer and its treatment may result in biological process such as the menopause occurring sooner or quicker, which could result in symptoms like fatigue, mood changes and sleep disturbance that are similar to those already experienced by cancer and possibly add further to them. This review has shown some evidence that may help women to manage these symptoms however, it has also demonstrated the need for more research in female specific cancers. Other reviews have also suggested the need for more research regarding race, ethnic group and types of cancer.^34^

Currently, nearly all the studies within this review were performed face to face, but as this review shows, adaptations to types and duration of mindfulness interventions did not have a detrimental effect on the overall effect, which may make it feasible to further adapt this type of intervention to a digital platform. Covid‐19 has resulted in a trigger for the use of online services to manage health and has been received in the most part positively however, it still has its issues such as poor bandwidth and users technology skills.^76^ Never the less the delivery of mindfulness interventions through these types of platforms would be worth further investigations as it would permit scalability and the ability to reach a larger population especially those in hard to reach rural area.^77^ Alongside this, the need for more robust studies with active comparators such as exercise that focus on outcomes such as CRF are needed.

Characteristics of mindfulness interventions particularly the type and duration did not demonstrate within this review a difference between groups. Although there appeared to be a slightly larger effect for the non‐MBSR and the less than 8 weeks in duration group all types of mindfulness demonstrated a favourable response to the outcomes of CRF, anxiety and depression. Cillessen et al., 2019,^72^ also found that there was no difference in efficacy between types of mindfulness intervention and that all types were effective however, they did establish that there was a larger effect if adherence to the original protocol was maintained. These results may demonstrate that reducing the length of the intervention or adapting the type of mindfulness does not appear to have a detrimental effect on the overall effect and indeed may aid delivery in future clinical settings, as compliance may be easier with shorter interventions. This would also have financial implications for clinical settings and possibly reduce participant burden.

When assessing types of comparator, the analysis showed there were no differences between sub groups the comparison against WLC appears to show a greater favourable response in this review for all outcomes, which has also been identified in other reviews. This is not unexpected as studies that have a WLC or UC can result in a larger effect size in the intervention arms.^34^ The small number of studies that used AC as the control arm in this review (*n* = 6, 32%) may have contributed to the findings. Other reviews also found similar findings where there were either too few studies to perform sub group analysis or the results were more favourable for the intervention groups compared to an AC group.^31^, ^34^ Furthermore, the type of AC may have an effect, as other reviews have suggested that if the comparator interventions are not developed for the management of particular symptoms, such as fatigue, then the results for these outcomes may not be reflective.^34^ The use of specific interventions for control arms was demonstrated by Monti 2013 et al., in their 3 arm study with women with breast cancer and the effect of mindfulness or educational support on stress and QoL. They found that even though the MBAT maybe more beneficial for stress reduction and improved QoL than the support group or untreated group, the active control group was still beneficial and therefore still a worthy intervention in this population. Alongside this comparing or combining mindfulness with other interventions such as exercise^78^ which is already supported by guidelines for managing fatigue, may provide evidence to enable health care professionals to make informed decisions with regard to interventions that could be offered to their patients.

## 5 CONCLUSION

In conclusion, there appeared to be a large effect of mindfulness on fatigue, anxiety and depression however, there was little or no effect on sleep and QoL. In terms of sub group analysis, there was little difference in the groups suggesting that all types of mindfulness and duration of intervention were effective. Follow up showed that the response was sustained although slightly reduced when compared to post intervention which may indicate the need for participants to continue practising. The demographics of the participants reported within the studies in this review show that certain types of cancer and ethnicity of the participants are underrepresented and further studies may aim to address this. Overall mindfulness is an intervention that is well received with few adverse events and is adaptable, which will make it a transferrable and scalable intervention within the clinical setting.

## AUTHOR CONTRIBUTIONS

All authors contributed and approved the final manuscript.

## CONFLICT OF INTEREST

The authors report no conflict of interest.

## ETHICS STATEMENT

For this type of study formal ethical approval was not required.

## Supporting information

Supporting Information S1

## Data Availability

Data sharing is not applicable to this article as no new data were created or analysed in this study.
