# Supplementary material for: Effects of mindfulness‐based interventions on fatigue and psychological wellbeing in women with cancer: A systematic review and meta‐analysis of randomised control trials
Source: Psychooncology. 2022 Oct 13;31(11):1821–34. doi: 10.1002/pon.6046 (PMC9828570; doi:10.1002/pon.6046)
Supplement: Supplementary file 1 — Supporting Information S1 [file PON-31-1821-s001.docx]

Search Strategy

Ovid Medline

1. Neoplasms/ (MeSH)

2. Cancer* or carcinoma* or tumo?r* or onco* or malignan* or neoplas* or metasta*

3. 1 or 2

4. Mindfulness/ (MeSH)

5. mindful* or mindfulness-based stress reduction or mindfulness-based cognitive therapy or mindfulness-based cancer recovery or MBSR or MBCT or MBCR or mindfulness-based intervention* or MBI or meditation

6. 4 or 5

7. fatigue/ (MeSH)

8. fatigue* or tired* or exhaust* or letharg* or weary or weariness or cancer-related fatigue* or CRF

9. 7 or 8

10. 3 and 6 and 9

11.Limit 10 to English language

EBSCO Cumulative Index to Nursing and Allied Health Literature (CINAHL)

S1. Neoplasms (MeSH)

S2. Cancer*

S3. Carcinoma*

S4. Tumo?r*

S5. Onco*

S6. Malignan*

S7. Neoplasm*

S8. Metasta*

S9. S1 or S2 or S3 or S4 or S5 or S6 or S7 or S8

S10. Mindfulness (MeSH)

S11. “Mindfulness-based stress reduction”

S12. “Mindfulness-based cognitive therapy”

S13. MBSR

S14. MBCT

S15. MBCR

S16. “mindfulness-based intervention*”

S17. MBI

S18. Meditation

S19. S10 or S11 or S12 or S13 or S14 or S15 or S16 or S17 or S18

S20. Cancer fatigue (MeSH)

S21. Fatigue*

S22. Tired*

S23. Letharg*

S24. Weary

S25. “cancer-related fatigue*”

S26. CRF

S27. S20 or S21 or S22 or S23 or S24 or S25 or S26

S28 S9 and S19 and S27

S30 English language limits on S2

| Table S1 | Sample size | | | | Effect Size^a^ | | | | | | Sub group difference | Heterogeneity^b^ | | | |
| --- | --- | --- | --- | --- | --- | --- | --- | --- | --- | --- | --- | --- | --- | --- | --- |
| Adapted types of mindfulness (non-MBSR v MBSR) | | | | | | | | | | |  |  | | | |
|  | MBSR | | Non MBSR | | MBSR | | | Non-MBSR | | | P | MBSR | | Non-MBSR | |
| Outcome | k | n | k | n | P | SMD | CI | P | SMD | CI |  | P | I² | P | I² |
| Fatigue | 8 | 919 | 8 | 414 | **0.006** | -0.62 | -1.06, -0.17 | **0.009** | -1.04 | -1.82,  -0.25 | 0.36 | <0.00001 | 88% | <0.00001 | 92% |
| Anxiety | 3 | 639 | 4 | 378 | **0.006** | -0.27 | -0.46, -0.08 | **0.006** | -1.47 | -2.51,  -0.42 | **0.03** | 0.23 | 31% | <0.00001 | 95% |
| Depression | 4 | 765 | 7 | 417 | **0.01** | -0.19 | -0.33, -0.04 | **<0.0001** | -1.30 | -1.91,  -0.68 | **0.0006** | 0.88 | 0% | <0.00001 | 87% |
| Sleep | 4 | 532 | 1 | 65 | **0.003** | -0.26 | -0.43, -0.09 | **<0.00001** | -2.88 | -3.58,  -2.17 | **0.00001** | 0.45 | 0% | N/A | N/A |
| QOL | 5 | 572 | 5 | 353 | 0.21 | 0.24 | -0.13, 0.60 | 0.36 | 0.53 | -0.61, 1.66 | 0.63 | 0.02 | 66% | <0.00001 | 96% |
| Length of intervention (8 weeks v < 8 weeks) | | | | | | | | | | |  |  | |  | |
|  | 8 weeks | | <8 weeks | | 8 weeks | | | <8 weeks | | |  | 8 weeks | | < 8 weeks | |
| Outcome | k | n | k | n | P | SMD | CI | P | SMD | CI |  | P | I² | P | I² |
| Fatigue | 11 | 856 | 4 | 482 | **0.009** | -0.56 | -0.98, -0.14 | **0.03** | -1.27 | -2.40, -0.14 | 0.25 | <0.00001 | 87% | <0.00001 | 96% |
| Anxiety | 5 | 592 | 1 | 311 | **0.02** | -0.78 | -1.46, -0.11 | **0.006** | -0.31 | -0.54, -0.09 | 0.19 | <0.00001 | 93% | N/A | N/A |
| Depression | 6 | 620 | 4 | 538 | **0.007** | -0.60 | -1.03, -0.16 | **0.03** | -0.86 | -1.62, -0.11 | 0.55 | <0.00001 | 84% | <0.00001 | 93% |
| Sleep | 2 | 157 | 3 | 440 | 0.80 | -0.09 | -0.83, 0.64 | 0.08 | -1.08 | -2.29, 0.14 | 0.17 | 0.11 | 62% | <0.00001 | 96% |
| QOL | 7 | 539 | 2 | 362 | 0.02 | 0.50 | 0.09, 0.90 | 0.32 | -0.90 | -2.68, 0.89 | 0.14 | 0.0003 | 76% | <0.00001 | 97% |
| WLC v ACC | | | | | | | | | | |  |  | |  | |
|  | AC | | WLC | | AC | | | WLC | | |  | AC | | WLC | |
| Outcome | k | n | k | n | P | SMD | CI | P | SMD | CI |  | P | I² | P | I² |
| Fatigue | 5 | 406 | 11 | 956 | 0.16 | -0.44 | -1.06, 0.18 | **<0.0001** | -1.06 | -1.57, -0.54 | 0.14 | <0.00001 | 87% | <0.00001 | 92% |
| Anxiety | 4 | 408 | 3 | 609 | 0.08 | -0.89 | -1.89, 0.10 | **0.03** | -0.95 | -1.79, -0.11 | 0.93 | <0.00001 | 95% | <0.00001 | 95% |
| Depression | 3 | 357 | 8 | 825 | 0.25 | -0.12 | -0.33, 0.09 | **<0.0001** | -1.14 | -1.67, -0.60 | **0.0005** | 0.93 | 0% | <0.00001 | 91% |
| Sleep | 2 | 157 | 3 | 440 | 0.80 | -0.09 | -0.83, 0.64 | 0.08 | -1.08 | -2.29, 0.14 | 0.17 | 0.11 | 62% | <0.00001 | 96% |
| QOL | 3 | 150 | 7 | 775 | 0.26 | 0.44 | -0.32, 1.20 | 0.31 | 0.34 | -0.32, 0.99 | 0.84 | 0.03 | 71% | <0.00001 | 94% |

**Table S1**

Abbreviations QOL, Quality of life; WLC, wait list control; ACC, active comparator control.

Bold figures indicate statistical significance.

^a^ ES= SMD, fatigue, anxiety, depression and sleep negative values indicates a favourable response. For QOL a positive value indicates a positive result. Values: small (0.2-0.5), moderate (0.5-0.8), large (>0.8).

^b^ P values < .1 taken to suggest heterogeneity. I² statistics: 0% (no heterogeneity), 25 % (low heterogeneity), 50% (moderate heterogeneity), and 75% (high heterogeneity).

| **Table S2 Summary of findings:** | | | | | | |
| --- | --- | --- | --- | --- | --- | --- |
| **Mindfulness compared to Control for women with cancer** | | | | | | |
| **Patient or population:** women with cancer  **Setting:**  **Intervention:** Mindfulness  **Comparison:** Control | | | | | | |
| Outcomes | **Anticipated absolute effects^*^** (95% CI) | | Relative effect (95% CI) | № of participants (studies) | Certainty of the evidence (GRADE) | Comments |
|  | **Risk with Control** | **Risk with Mindfulness** |  |  |  |  |
| Fatigue | - | SMD **0.87 lower** (1.27 lower to 0.46 lower) | - | 1362 (16 RCTs) | ⨁◯◯◯ Very low^a,b,c^ |  |
| Psychological well being (depression) | - | SMD **0.81 lower** (1.19 lower to 0.43 lower) | - | 1182 (11 RCTs) | ⨁◯◯◯ Very low^a,c,d^ |  |
| Psychological well being (anxiety) | - | SMD **0.92 SD lower** (1.5 lower to 0.3 lower) | - | 1017 (7 RCTs) | ⨁⨁◯◯ Low^a,e^ |  |
| Quality of Life | - | SMD **0.37 SD higher** (0.13 lower to 0.87 higher) | - | 925 (10 RCTs) | ⨁◯◯◯ Very low^a,c,f^ |  |
| Sleep | - | SMD **0.65 SD lower** (1.34 lower to 0.04 lower) | - | 597 (5 RCTs) | ⨁⨁◯◯ Low^a,g^ |  |
| ***The risk in the intervention group** (and its 95% confidence interval) is based on the assumed risk in the comparison group and the **relative effect** of the intervention (and its 95% CI).  **CI:** confidence interval; **SMD:** standardised mean difference | | | | | | |
| **GRADE Working Group grades of evidence** **High certainty:** we are very confident that the true effect lies close to that of the estimate of the effect. **Moderate certainty:** we are moderately confident in the effect estimate: the true effect is likely to be close to the estimate of the effect, but there is a possibility that it is substantially different. **Low certainty:** our confidence in the effect estimate is limited: the true effect may be substantially different from the estimate of the effect. **Very low certainty:** we have very little confidence in the effect estimate: the true effect is likely to be substantially different from the estimate of effect. | | | | | | |

#### Explanations

a. Most studies reported details on random sequence generation, however concealments was not clear in nearly half of the studies. Due to the nature of these studies blinding of participants and study personal was not possible. Majority of studies did not describe the blinding of assessors. the vast majority dealt with missing outcome data adequately. The selective reporting was difficult to assess as not reported resulting in bias.

b. There was evidence of large heterogeneity for changes in fatigue (p=<0.00001, I²=91%). Most of the confidence intervals overlapped there were 4 outliers.

c. Funnel plot demonstrated asymmetry strongly suggesting publication bias.

d. There was evidence of large heterogeneity for changes in depression (p=<0.00001, I²=89%).

e. There was evidence of large heterogeneity for changes in anxiety (p=<0.00001, I²=95%).

f. There was evidence of large heterogeneity for changes in quality of life (p=<0.00001, I²=91%).

g. There was evidence of large heterogeneity for changes in sleep (p=<0.00001, I²=92%). Most of the confidence intervals overlapped , there was one study outlier.

Figure S1 Funnel plots for publication bias


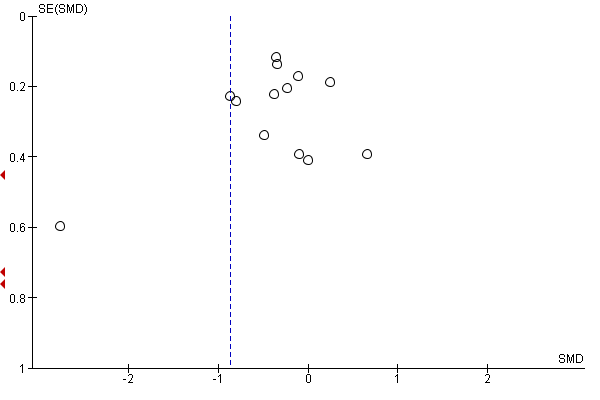


Funnel plot for fatigue for publication bias


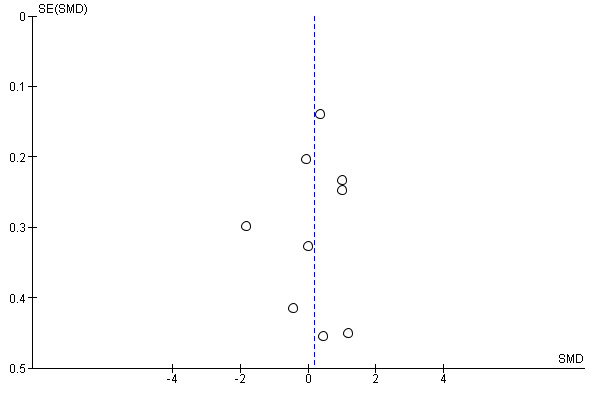


Funnel plot for QoL for publication bias
